# The Season for Renewed Effort

**Published:** 1900-10

**Authors:** 


### Editorial.

### THE SEASON FOR RENEWED EFFORT.

In this country, owing to climatic influences, there is a season of activity and one that nearly borders on stagnation, a period beginning with June and ending with September. That this follows laws of cause and effect is quite true, having no special relation to man and his needs. It is, however, equally true that but for this period of repose the nervous activities engendered by these conditions would wear out the strongest organization. While all business languishes during this hot period, it is a time well adapted for reflection and the formation of plans for the approaching active season.

The period of partial repose of all the activities, mental and physical, is usually the one selected by the dental fraternity to hold the national and State conventions. That this selection of time is open to criticism needs no argument, but it will, probably, remain as originally selected for years to come. It must be apparent that an earlier period in the year—May or early June—would be productive of greater results. Calling men together with the thermometer ranging from 80° to 95° F. is not conducive to the display of superior mental ability. The effect of this was manifest at all the conventions held during this hot summer. It is, however, useless to expect any change, for those who feel it a duty and a pleasure to attend these meetings will rather bear the physical torture than the loss of practice at a more comfortable period. While this is, apparently, a finality in the dentistry of this country, it may be left where it remains and attention be given to problems more difficult of solution.

The general convention period having closed, the interest will soon be awakened to the work of the local societies. These are the true sources of dental progress. The national and State organizations broaden social professional life, but they add little to the sum of general dental knowledge. This must, necessarily, have its source, first, in individuals, and then from these to local societies. When the work of the latter bodies is compared with that of the National Dental Association, the latter suffers by comparison. This should be expected, as the individual is in closer touch with the local body and receives his enthusiasm for work from this more nearly organized effort.

This brings up the thought and the question, Is there a sufficiently well devised plan as a basis for work in these local organizations? In looking over the subjects brought before these societies, the mind is impressed with the fact that papers are written and accepted without much attention being paid to their intrinsic value. The object of the Society Committee seems to be to secure a programme, very often regardless of quality. The result is, therefore, of little value from a scientific stand-point. It is a waste of time to thresh over old subjects, and it is wearisome to read of "Methods of Filling Teeth," "The Value of Soft-gold as compared with Cohesive," "The Treatment of Pulp-Canals," etc., etc. If the dental profession has not been able to settle these questions through an experience of a century of work, they might be left until it reaches a period when some minds will rise beyond rudimentary thought and give a clear and positive answer to conflicting theories. These questions have, however, long ago been practically settled, but the rising generation of dentists will not have it so, and, ignoring the experience of the past, persist in burdening societies with their crude conceptions of what ought to be legitimate practice. It must not be thought that objection is made to the thorough consideration of matters connected with the subjects named and that of many others. There is a way that leads to truth, and there is another that confirms ignorance. If a paper shows that the author has experimented days and months to find a better way to practise, he is entitled to a respectful hearing, but he is bound to demonstrate that he is at the same time familiar with all that has preceded his effort in the history of dentistry.

The National Dental Association has started in the right direction to secure better results, and the outcome will be looked for with interest. Whether it accomplishes much or little, it should still be an example for local organizations to follow. The older dental societies did much better than many at present. When a problem for solution was presented, it was usual to select a committee of the best and most original workers to examine the subject thoroughly and report. Within the writer's knowledge some of these spent months in careful work. In this way the Pennsylvania Association of Dental Surgeons settled two very important questions,—first, as to the value of "sponge gold" as a filling-material, and, second, whether free mercury existed in rubber plates, resulting in an inflamed mucous membrane. The conclusion in regard to sponge gold, which covers practically all the more recent forms, has never been successfully disputed, and the latter subject was so thoroughly examined, chemically and microscopically, that the last word was said then and there in regard to mercury producing the pathological condition named. It was left, however, for Dr. Black to furnish the key that opened the door to a final escape from this troublesome feature in this class of dentures.

These facts are simply illustrative of the true in opposition to the false method. The one seeks to know, while the other assumes to know. The one laboriously gropes for knowledge, step by step, while the other takes up the pen and theorizes, brilliantly, it may be, on paper. Societies need the former method. It is feared, however, that the same wearisome platitudinous round of words will be continued unless societies use different methods than those at present in vogue. It will be uplifting if in the near future some dental organization will begin a year in advance to portion out unsolved problems to be reported upon when a sufficient number of facts have been obtained to furnish a basis for conclusions.

It is noted with pleasure that individual work in original investigation has been steadily growing the past few years, doubtless due to that higher training given in all the dental schools. Young men are receiving an incentive for future usefulness, and it is to be hoped that the objectionable essay alluded to will soon become a thing of the past.

Whether this winter, or winters succeeding, will show a change in method in society work, the trend of thought and practice is all pointing in the direction outlined, and we may expect in the not far distant future that papers will be made up from observed facts, and discussions will be limited to the subject of the essay, a condition of affairs most earnestly desired by all who prefer truth to theory, and condensed thought to a multiplication of words.

